# Use of Janus kinase inhibitors before and after European Medicines Agency safety recommendations: a retrospective study

**DOI:** 10.3389/fimmu.2024.1445680

**Published:** 2024-08-22

**Authors:** Patrick-Pascal Strunz, Linus Maximilian Risser, Matthias Englbrecht, Torsten Witte, Matthias Froehlich, Marc Schmalzing, Michael Gernert, Sebastian Hueper, Peter Bartz-Bazzanella, Cay von der Decken, Kirsten Karberg, Georg Gauler, Susanna Späthling-Mestekemper, Christoph Kuhn, Wolfgang Vorbrüggen, Martin Welcker, Stefan Kleinert

**Affiliations:** ^1^ University Hospital of Würzburg, Department of Medicine II, Rheumatology/Clinical Immunology, Würzburg, Germany; ^2^ Medical School Hannover, Department of Rheumatology and Immunology, Hannover, Germany; ^3^ Freelance Healthcare Data Scientist, Greven, Germany; ^4^ Praxis für Rheumatologie und Osteologie, Hildesheim, Germany; ^5^ Klinik für Internistische Rheumatologie, Rhein-Maas-Klinikum, Würselen, Germany; ^6^ Medizinisches Versorgungszentrum, Stolberg, Germany; ^7^ RHADAR – RheumaDatenRheport GbR, Planegg, Germany; ^8^ Verein zur Förderung der Rheumatologie e.V., Würselen, Germany; ^9^ Rheumatologisches Versorgungszentrum Steglitz, Berlin, Germany; ^10^ Rheumatology Practice, Osnabrück, Germany; ^11^ Rheumapraxis München, Munich, Germany; ^12^ Praxis für Rheumatologie, Karlsruhe, Germany; ^13^ Medizinisches Versorgungszentrum für Rheumatologie Dr. M. Welcker GmbH, Planegg, Germany; ^14^ Praxisgemeinschaft Rheumatologie-Nephrologie, Erlangen, Germany

**Keywords:** rheumatoid arthritis, cancer, treatment, tumor necrosis factor inhibitors, interleukin-6 receptor inhibitors, oral surveillance, major adverse cardiac event, venous thromboembolism

## Abstract

**Background:**

Safety recommendations for Janus kinase inhibitors (JAKi) issued by the European Medical Agency (EMA) in 2023 could potentially influence treatment patterns for rheumatoid arthritis (RA) drugs, but little is known about the impact of these recommendations in routine clinical care.

**Methods:**

We retrospectively analyzed the German RHADAR rheumatology database for adult patients with RA and documentation of a new therapy with a JAKi, tumor necrosis factor inhibitor (TNFi), or interleukin-6 receptor inhibitor (IL-6Ri). Data were grouped into half-yearly intervals from quarter (Q)2/2020 to Q3/2023. The period from Q4/2022 to Q1/2023 immediately followed the initial EMA endorsement of Pharmacovigilance Risk Assessment Committee (PRAC) recommendations and Q2/2023-Q3/2023 immediately followed the direct healthcare provider communication (DHPC) containing the new safety JAKi recommendations.

**Results:**

Between April 1, 2020 and September 23, 2023, 3008 newly initiated therapies for TNFi (1499 [49.8%]), JAKi (1126 [37.4%]), and IL-6Ri (383 [12.7%]) were documented by the treating physicians. JAKi were increasingly used in the first two half-year periods (from 29.7% of these therapies in Q2/2020-Q3/2020 to 46.7% in Q2/2021-Q3/2021; odds ratio [OR] 2.08; p<0.001). The proportion of initiated JAKi therapies decreased significantly after the PRAC recommendations (32.9%; OR vs peak 0.56; p=0.001) and the DHPC letter (26.1%; OR vs peak 0.40; p<0.001). JAKi were more likely to be used as >3^rd^-line therapy in later time periods.

**Conclusions:**

This exploratory study suggests that EMA safety recommendations for JAKi influenced treatment patterns of RA patients who received JAKi in Germany. Additional studies will be needed to confirm these findings.

## 1 Introduction

Janus kinase inhibitors (JAKi) play a key role in the management of chronic immune-mediated disorders. Tofacitinib was approved by the European Medicines Agency (EMA) in 2017 for rheumatoid arthritis (RA) and was soon joined by other drugs in this class, including baricitinib, upadacitinib, and filgotinib. Although these agents all affect the JAK/STAT pathway, they vary in their selectivity for different kinases and cell types (1).

The manufacturers of tofacitinib were required to conduct a post-approval safety trial. The resulting ORAL Surveillance Study, which was published in January 2022, found that tofacitinib was associated with significantly higher rates of major adverse cardiovascular events, cancers, and opportunistic infections compared with tumor necrosis factor inhibitors (TNFi) in RA patients ≥50 years with ≥1 cardiovascular risk factor (2). Based on these data, regulatory agencies strengthened safety recommendations for all JAKi. On November 11, 2022, the EMA endorsed recommendations from the Pharmacovigilance Risk Assessment Committee (PRAC) stating that JAKi should only be used if no suitable treatment alternatives are available in patients 65 years or older, at increased risk of major cardiovascular problems or cancer, or current or long-term past smokers, and should be used with caution in patients at risk for venous thromboembolism (3). A direct healthcare professional communication (DHPC) with this information was sent to clinicians in Europe on March 30, 2023 (4).

The effect of these regulatory changes on rheumatology treatment practices has not been well studied. We used the German RHADAR rheumatology database (5) to assess the proportions of patients initiating therapy with JAKi, TNFi, or an interleukin-6 receptor inhibitor (IL-6Ri) across time periods spanning before and immediately after the regulatory changes.

## 2 Methods

### 2.1 Study design

This exploratory study was a non-interventional, retrospective analysis of pseudonymized data collected during routine clinical care from adult patients (≥18 years) in the RheumaDatenRhePort (RHADAR) GbR (A Network of Rheumatologists) database, which includes patients seen at German clinical rheumatology sites (5).

Data were evaluated in half-year intervals (two quarters ([Q]; Q2–Q3=April 1 to September 30; Q4–Q1=October 1 to March 31) over the following time periods:

- Before PRAC recommendations: Q2/2020–Q3/2020; Q4/2020–Q1/2021; Q2/2021–Q3/2021; Q4/2021–Q1/2022; Q2/2022–Q3/2022
- After PRAC recommendations: Q4/2022–Q1/2023
- After DHPC: Q2/2023–Q3/2023 (ending September 23, 2023)

All patients gave prior written informed consent for evaluation within the RHADAR database. Included patients had a diagnosis of RA and documentation of the initiation of treatment with a JAKi, TNFi, or IL-6Ri between April 1, 2020 and September 23, 2023; no other selection criteria were applied. Data on patient characteristics and therapies were collected as part of routine care.

A draft of the study was submitted to the Ethics Committee of the Hannover Medical School (Application 11291_BO_K_2024), which advised that the retrospective analysis of anonymized clinical data did not require ethical approval according to German law.

### 2.2 Outcomes

We evaluated patient characteristics, initiation of specified new treatments, and line of therapy as included in patient documentation during routine clinical care. Relevant comorbidities related to the PRAC/EMA safety recommendations (cardiovascular risk factors and malignancy) were based on ICD-10 codes and included comorbidities recorded in the database at any point in time.

### 2.3 Statistical analysis

A study size calculation was not performed due to the retrospective nature of the study. Sample size was determined by all available prescriptions in the registry during the observational period.

We analyzed descriptive data for patients with reported data; missing data were not imputed. For sample characterization, we used absolute and relative frequencies and means with standard deviation (SD). To evaluate changes in the proportion of JAKi therapies over time, we used odds ratios (OR) and 95% confidence intervals (CIs) with adjusted p values derived by the Benjamini-Hochberg method (6). Statistical significance was set at p ≤ 0.05. Statistical analyses were performed using R software (version 4.3.0), RStudio (version 1.1.453), and Prism Version 5.

## 3 Results

### 3.1 Initiation of new therapies and patient characteristics

Between April 1, 2020 and September 23, 2023, 3008 new therapies with a JAKi, TNFi, or IL6-Ri were documented in the RHADAR database. The TNFi drug class was initiated most frequently (1499 [49.8%]) followed by JAKi (1126 [37.4%]) and IL-6Ri (383 [12.7%]). Baseline characteristics and comorbidity profiles by drug class were generally similar (**Table 1**). Patients receiving treatment with TNFi were slightly younger (mean of 56.6 years) than those receiving treatment with JAKi (60.4 years) or IL-6Ri (61.8 years) and had a shorter disease duration (mean of 11.7 years) than those treated with JAKi (13.7 years).

**Table 1:** Patient characteristics at time of therapy initiation.

Characteristic	JAKi	TNFi	IL-6Ri
Number of new therapies	1126	1499	383
Female, n/N (%)	915/1126 (81.3%)	1190/1499 (79.4%)	300/383 (78.3%)
Age, mean years (SD) [n]	60.4 (13.2) [n=1124]	56.6 (15.4) [n=1496]	61.8 (13.7) [n=382]
Disease duration, mean years (SD) [n]	13.7 (10.2) [n=1028]	11.7 (10.1) [n=1289]	11.5 (9.2) [n=336]
RF positive, n (%)	713 (63.3%)	838 (55.9%)	204 (53.3%)
Selected comorbidities,^a^ n (%)			
Missing data	39 (3.5%)	74 (4.9%)	14 (3.7%)
No selected comorbidities	8 (0.7%)	26 (1.7%)	4 (1.0%)
Diabetes	73 (6.5%)	89 (5.9%)	37 (9.7%)
Hypertensive disease	352 (31.3%)	389 (26.0%)	139 (36.3%)
Vein thrombosis	10 (0.9%)	27 (1.8%)	10 (2.6%)
Pulmonary embolism	5 (0.4%)	12 (0.8%)	4 (1.0%)
Coronary heart disease	107 (9.5%)	104 (6.9%)	40 (10.4%)
Angina pectoris	0	0	0
Myocardial infarction and complications	2 (0.2%)	1 (0.1%)	1 (0.3%)
Stroke	25 (2.2%)	24 (1.6%)	9 (2.3%)
Hyperlipidemia/hypercholesterolemia	164 (14.6%)	166 (11.1%)	49 (12.8%)
Nicotine abuse	42 (3.7%)	60 (4.0%)	16 (4.2%)
Malignancies	50 (4.4%)	45 (3.0%)	22 (5.7%)

a Based on comorbidities recorded for the patient at any time point before or after therapy initiation.

IL-6Ri, interleukin-6 receptor inhibitor; JAKi, Janus kinase inhibitor; RF, rheumatoid factor; SD, standard deviation; TNFi, tumor necrosis factor inhibitor; VAS, visual analog scale.

### 3.2 Changes in treatment patterns over time

**Figure 1:**
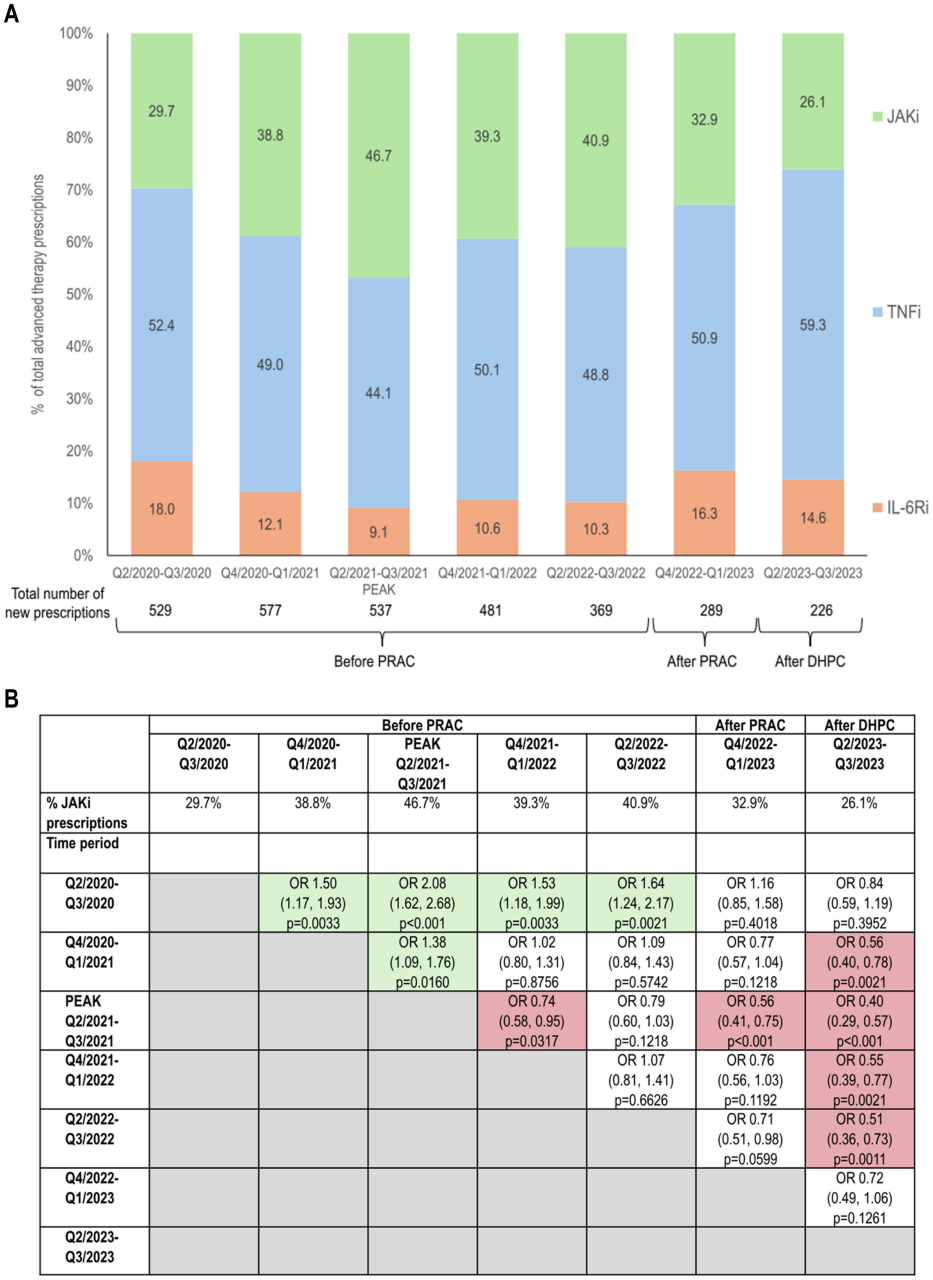
Changes in initiation of JAKi therapy in the time periods before and after JAKi EMA safety recommendations. **(A)** Proportion of new therapies initiated with JAKi, TNFi, or IL-6Ri; **(B)** Odds ratios (95% CI) and statistical significance of changes in proportions of newly initiated JAKi treatment over time. Green shading indicates significant increases in the proportion of JAKi therapies and red shading indicates significant decreases in the proportion of JAKi therapies. P values were calculated using the Benjamini-Hochberg adjustment. CI, confidence interval; DHPC, direct healthcare provider communication; EMA, European Medical Agency; IL-6Ri, interleukin-6 receptor inhibitor; JAKi, Janus kinase inhibitor; PRAC, Pharmacovigilance Risk Assessment Committee recommendations; Q, quarter; TNFi, tumor necrosis factor inhibitor.

The proportion of patients initiating therapy with JAKi significantly increased from 29.7% in the earliest period (Q2/2020-Q3/2020) to a peak of 46.7% one year later (Q2/2021/Q3/2021; OR 2.08 [95% CI 1.62, 2.68]; p<0.001) and then decreased, particularly in the post-PRAC (32.9%; Q4/2022-Q1/2023; OR vs peak 0.56 [95% CI 0.41, 0.75]; p<0.001) and post-DHPC (26.1%; Q2/2023-Q3/2023; OR vs peak 0.40 [95% CI 0.29, 0.57]; p<0.001) periods (**Figure 1**). The proportion of newly initiated TNFi therapies showed the opposite pattern (decreases followed by increases). The proportions of new treatments with IL-6Ri showed modest variability over the observation period (**Figure 1**).

Numerically, the Q4/2020-Q1/2021 time period had the most newly initiated therapies for these three drug classes (total of 577) and numbers of new therapies fell throughout the remaining periods (**Figure 1**).

**Figure 2:**
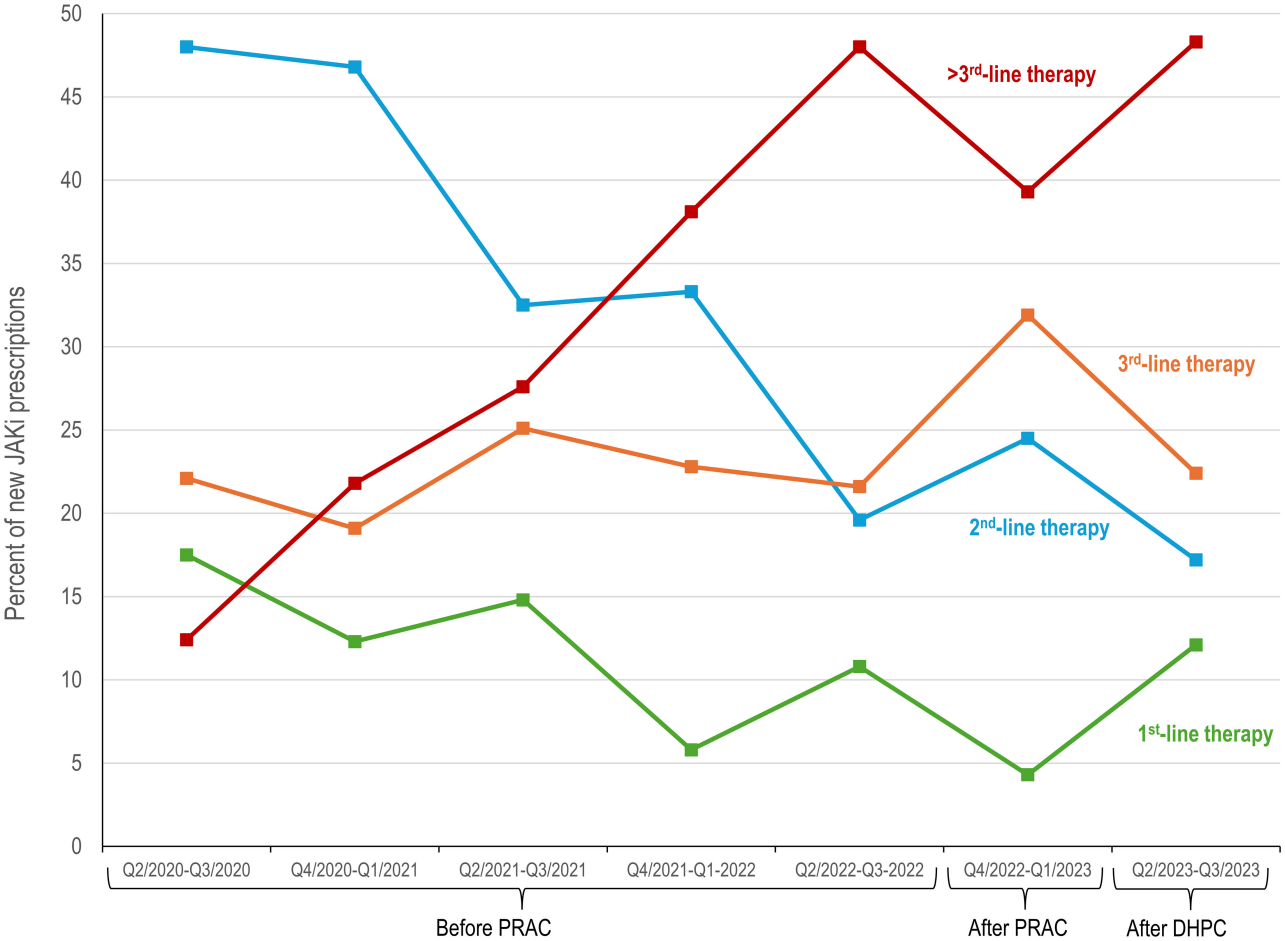
Changes in line of therapy in patients initiating JAKi treatment before and after JAKi EMA safety recommendations. DHPC, direct healthcare provider communication; EMA, European Medical Agency; JAKi, Janus kinase inhibitor; PRAC, Pharmacovigilance Risk Assessment Committee recommendations.

New JAKi usage as 1^st^-line therapy generally decreased over time, although there was variability across time periods (**Figure 2**). Use as 2^nd^-line therapy decreased markedly (from 48.0% Q2/2020-Q3/2020 to 17.2% Q2/2023-Q3/2023), and this was accompanied by large increases in use as >3^rd^-line therapy (from 12.4% to 48.3%).

## 4 Discussion

The data from this exploratory analysis provide insights into changes in treatment practices in RA patients following the issuance of new safety recommendations for JAKi. Compared with earlier time periods, there was a significant decrease in the proportions of patients receiving new JAKi treatment in the time periods following the safety recommendations, and JAKi therapies initiated later in the study were more likely to be used as >3^rd^-line therapy.

In our study, the proportion of new JAKi therapies peaked during the Q2/2021-Q3/2021 time period. The decreases observed in the two following half-year periods, which were prior to the PRAC and DHPC time points, suggest that professionals may have begun to consider the implications of JAKi safety data on patient selection prior to the EMA recommendations; preliminary data were released by the manufacturer in January 2021 (7) and subsequently presented at a November 2021 conference (8). The proportions of new TNFi therapies showed an inverse pattern to JAKi. Our findings are consistent with a US study of RA prescriptions, which found significant decreases in JAKi prescriptions after the January 2021 release of preliminary ORAL Surveillance data and an association between decreases in new JAKi prescriptions and increases in TNFi use (9).

In addition to rheumatologic conditions, the EMA safety recommendations apply to all other JAKi indications as well. In particular, JAKi are frequently used for dermatologic conditions, including psoriasis and atopic dermatitis. Increased rates of malignancy and thromboembolic events have been observed in dermatologic patients treated with systemic JAKi drugs (10), particularly older patients (11), who are more likely to have multiple risk factors (12). It would therefore be of interest to also evaluate JAKi prescription patterns in these populations.

To the best of our knowledge, the change in the line of therapy for JAKi drugs over time has not been reported elsewhere and does not seem to have a clear alternative explanation. This change in treatment patterns is likely explained by the fact that a large proportion of RA patients have characteristics or comorbidities specified in the JAKi safety recommendations, including age of 65 years or older (13, 14), and therefore would not be considered good candidates for 1^st^-line JAKi therapy based on EMA guidance.

An unavoidable confounding factor is that the earlier study time periods were likely affected by changes in treatment patterns and IL-6Ri shortages during the COVID-19 pandemic (15, 16). Somewhat surprisingly, we observed higher numbers of new advanced therapies during the pandemic period compared with later timepoints. The reasons for this remain unclear, but may relate to the specific drug classes evaluated in this study and pandemic-related changes in prescribing practices. In particular, rituximab and methotrexate showed decreases in drug initiation during the pandemic (17), likely due to concerns about poor COVID outcomes and reduced vaccination responses (18–20). It is possible that as treatment patterns normalized during post-pandemic years, some patients on the therapies evaluated here (JAKi, TNFi, and IL-6Ri) returned to treatment with drugs not included in our analyses. In addition, the delayed documentation frequently observed in EHR data may have artificially decreased numbers of documented therapies during later time periods. However, it is unlikely that the lower numbers of therapies in the last two time periods affected the proportions of drug usage observed in our study.

Study limitations include those inherent to retrospective database analyses, including the inability to verify treatment adherence. Some data, such as smoking status, were not routinely reported. Reasons for treatment discontinuation and comorbidity start dates were not available in our database. Other studies have noted decreases in JAKi-treated patients with cardiovascular risk factors and other relevant comorbidities (21–23). Our study was focused on a subset of advanced therapies, and inclusion of additional agents may have affected the treatment patterns observed here. In addition, our analyses were limited to patients with RA and did not include other rheumatology and non-rheumatology indications for JAKi, such as psoriatic arthritis or axial spondyloarthritis (24, 25).

In conclusion, our data suggest that the updated EMA safety recommendations for JAKi were associated with changes in treatment practices for patients with RA in Germany. Future studies will be required to see if these changes represent a temporary shift or more long-lasting usage patterns.

## Data Availability

The original contributions presented in the study are included in the article/supplementary material. Further inquiries can be directed to the corresponding author.
